# How do consumers respond when presented with novel doctor performance information? A multivariate regression analysis

**DOI:** 10.1111/hex.13378

**Published:** 2021-12-01

**Authors:** Michelle B. Hanson

**Affiliations:** ^1^ Free University of Bozen‐Bolzano Bolzano Italy

**Keywords:** decision‐making, doctor attributes, doctor choice, primary health care, quality ratings

## Abstract

**Background:**

There is an array of attributes one may consider when selecting a doctor. Consumers must generally select providers in the absence of any standardized performance information about these attributes at the doctor level. Some attributes may be less salient to consumers until presented with novel performance data. Innate decision‐making regret, style and skill may be important, given the complexity of processing and trading off on numerous attributes.

**Objective:**

There has been limited opportunity to study consumer behaviour in the presence of doctor‐level quality information, as these data are not widely available. This study explores how consumers interact with doctor‐level performance data, considering their decision‐making regret, style and skill. Specifically, it examines how consumers rate 10 doctor attributes before and after exposure to doctor‐level quality information.

**Methods:**

The study utilizes data from the SelectMD 2.0 Provider Choice Experiment. Respondents (*n *= 1247) were presented with a mock website reporting quality information and asked to choose a doctor. Difference scores are calculated based on participants' ratings of 10 attributes before and after the experiment and a multivariate ordered probit regression is considered to study the association between the predictors and 10 response outcomes.

**Results:**

Consumers change their valuation of doctor attributes following exposure to quality data. As expected, consumers upgrade their valuation of the safety and technical quality attributes, but this is specifically associated with a greater tendency to regret decisions. Instead, those with a more dependent decision‐making style downgrade reputation, while those with better decision‐making skill downgrade the bedside manner and safety attributes.

**Patient or Public Contribution:**

Consumers/patients participated in the pilot testing of the website used for the experiment.

## 1 INTRODUCTION

Performance transparency in health care is being advanced in the United States and other countries to better inform consumer choice and drive greater competition between health care providers to induce improved quality of care. Despite growing availability of health care quality reports, however, there is evidence that consumer awareness is still limited, and where there is awareness, it is still questionable whether there is the interest or ability to use this information functionally to make decisions.^1^, ^2^, ^3^, ^4^ There is much discussion as to why this is the case, but one important factor appears to be that the information available may not be a good match with the information that consumers want or need.^5^, ^6^ For primary care in the United States, standardized performance measures are generally reported at the clinic level in their most granular form; thus, consumers are left to choose their regular doctor without access to provider‐specific performance data.

Within a 12‐month period, it is estimated that 7.5% of US adults look for a new personal doctor.^7^ Translated to the current US population, this means that more than 24 million people may be searching for a new doctor during the course of any one year. This represents a considerable opportunity to provide data that can help consumers to find a doctor right for them based on their preferences.

There is a scarcity of research exploring consumer preferences and choice regarding primary care doctors. Of the 118 studies included in a broad scoping review of determinants for patient choice of providers conducted by Victoor et al.,^8^ only 12 studies were focused on primary care doctors. Studies of consumer preferences in choosing a doctor present a variety of results, dependent in part on what the study design can measure and what doctor characteristics are included.^8^, ^9^, ^10^, ^11^, ^12^, ^13^ Studies exploiting administrative data to determine revealed preferences have pointed to consumers choosing primary care doctors who resemble themselves in terms of observable characteristics, namely, age and gender.^11^, ^14^ Survey studies, such as that by Bornstein et al.,^15^ found that professional factors and management practices outweigh a doctor's personal characteristics, while another study by Kenny et al.^12^ indicated care quality as the most essential, where a summation scale broadly grouped together technical care, interpersonal care and continuity. Perrault and Inderstrodt‐Stephens^13^ found communication style as being most important, but in the absence of including any elements of technical quality. Discrete choice experiments have indicated that, for many, technical quality outweighs interpersonal skills, and yet a substantial proportion of respondents placed the highest value on interpersonal skills.^10^, ^16^

Notwithstanding these efforts in an attempt to understand consumer preferences and choice concerning primary care doctors, there has been limited opportunity to study these aspects in the presence of doctor‐level quality information, as these data are not widely available. Evidence from consumer choices made on experimental websites suggests that if doctor‐level quality data are made available, it has an impact on the choices that consumers make when selecting among clinicians.^17^, ^18^, ^19^ Here, the primary research question was how differing data presentations affect choice quality, with a principal finding that adding patient review comments leads to better engagement with the website, but distracts consumers from standardized quantitative measures and can lead to deteriorating decision quality for some subsets of consumers.^17^, ^18^ Further, consumers vary in their ability to absorb this information and appear to differ in their utilization of this information based on decision‐making style and skill.^19^

Next to nothing is known, however, about how exposure to this same doctor‐level quality information could affect consumers' preferences concerning primary care doctors. How does the ability to observe variation in clinician performance impact the way consumers valuate certain doctor attributes, particularly safety and technical quality, which are often assumed by consumers to be relatively consistent among providers?^20^, ^21^ This study offers the first insights into how consumers' expressed preferences change following exposure to novel performance metrics.

The study exploits data collected in the SelectMD 2.0 Provider Choice Experiment, which utilized a mock website reporting doctor‐specific performance data and required a choice task.^22^ The original study was funded by the US Agency for Healthcare Research and Quality to learn how to provide consumers with better information and support when choosing a doctor. As part of the study, participants rated 10 doctor attributes both before and after exposure to the website. Given that performance data relative to the attributes of safety and technical quality may be considered the most novel information presented, I hypothesize that consumers will upgrade the importance placed on these two attributes following exposure to the website (Hypothesis 1).

Further, the study tests how decision‐making regret, style and skill, determined in the postexperiment survey, are associated with shifts in valuation of the 10 attributes. It is anticipated that one's characteristic mode of approaching decision‐making, including absorbing information delineating choice alternatives, will have an effect on shifts in preferences. Russo et al.^23^ highlight decision‐making style as an important psychological construct relating to the formation of patient preferences, noting, however, that this has yet to be well investigated.

Decision regret is characterized by looking back with lingering doubt over a decision made or regretting having missed out on a different outcome.^24^ I hypothesize that those with a greater tendency towards decision regret will be more sensitive to data showing variation in safety and technical quality and thus be more likely to upgrade their valuation of the attributes (Hypothesis 2).

Four distinct decision‐making styles (avoidant, dependent, intuitive and rational) are evaluated based on Scott and Bruce's decision‐making inventory.^25^ As an avoidant decision‐maker will seek to evade decision‐making processes and those with an intuitive style tend to trust their gut, I hypothesize that these decision‐making styles will not influence much change in ratings across the attributes following exposure to the website (Hypothesis 3). Instead, for those with a more dependent decision‐making style, typically guided by the opinion of others, I anticipate that they will decrease the initial importance placed on the reputation attribute once presented with other information by which to make their decision (Hypothesis 4). Lastly, for rational decision‐makers, more likely to thoroughly and logically evaluate alternatives, I anticipate that they would find greater salience for the safety and technical quality attributes following exposure to the website and also upgrade their ratings for these two attributes (Hypotheses 5).

Finally, decision‐making skill is tested based on how well consumers can utilize standardized and quantified ratings of performance to make consumer decisions, based on a hypothetical (and non‐health‐related) consumer choice task.^26^ Those with better decision‐making capabilities are also expected to upgrade the safety and technical quality attributes more than those who find these comparisons more challenging, as the more skilled consumers should be more likely to recognize that providers have disparate performance on these attributes (Hypothesis 6).

## 2 METHODS

### 2.1 Data and experimental design

This study utilizes data generated by the SelectMD 2.0 Provider Choice Experiment, which was developed to study various methods of incorporating quantitative doctor performance data (measures of patient experience, clinical quality and patient safety) with individual patient review comments to facilitate consumer choice of doctor.^22^ Participants were recruited through a probability‐based internet survey panel designed to be representative of the US population. The original experiment was reviewed and approved by the RAND Human Subjects Protection Committee, RAND's Institutional Review Board (IRB), to review research involving human subjects. For an in‐depth description of the study protocol and sample characteristics, see Cerully et al.^22^

The experiment involved a choice task in which respondents were presented with a functional, mock website reporting quality information for a set of 12 fictional doctors and then asked to choose a (hypothetical) doctor. The seven experimental arms differed by which types of quality information were included and the way in which the measures were presented, with one arm incorporating the use of a patient navigator, a person available by phone to assist the respondent in navigating the website. Participants also completed a pre‐ and post‐survey that included questions related to respondents' preferences when choosing a doctor, their level of consumer activation and a number of dimensions measuring decision‐making regret, style and skill.

The study took place in stages beginning with recruitment and completion of the pre‐survey. One week later, participants were invited to return to the experiment and were linked to the SelectMD website, where they were presented with quality information (varying by experimental arm assigned) and asked to select a doctor. Immediately after selecting a doctor, respondents were directed to a post‐survey. One week was allowed to elapse between pre‐ and post‐surveys to reduce the chance that respondents would strive to maintain consistency in answering the questions that were repeated measures, such as the attribute ratings.

The present study capitalizes on these pre‐ and post‐survey data to examine whether or not respondents shift their ratings of 10 doctor attributes following exposure to the quality‐reporting website. In the pre‐survey, respondents were asked to rate each of 10 attributes in terms of how much they mattered to them when selecting a primary care doctor. The response scale was as follows: 1 = not matter much; 2 = matter some; and 3 = matter a lot. The list of attributes was presented in random order and is shown in Table 1 along with the short description provided to the respondents. In the post‐survey, respondents were again presented with the same 10 attributes, this time with a query as to how much each attribute mattered when they selected a doctor on the website. While the performance measures reported on the website were not an exact one‐to‐one match with the 10 doctor attributes rated by the respondents, the majority of the attributes mapped to the three general categories of performance measures, which were Use of Effective Treatments, Methods to Reduce Medical Errors and Patient Survey Results.

**Table 1:** Doctor attributes rated by respondents in pre‐ and post‐surveys, with a short description provided to the respondents

Attribute	Description
Bedside manner	Warmth, caring, good listener
Office staff	Friendly, courteous, helpful
Patient complaints	Few complaints or malpractice charges
Proximity	Close to home or work
Reputation	Recommended by others
Safety	Avoiding medical errors
Technical quality	Gives patients best treatments and tests
Time availability	Not rushed during visits
Treatment orientation	How aggressively treats illness
Trustworthy	Choices on behalf of the patient, not insurer

Decision‐making regret, style and skill were determined in the post‐experiment survey. Respondents were asked to what degree (on a 5‐point Likert scale) they agreed or disagreed with five items from the Schwartz et al. regret scale.^24^ These responses are aggregated to create one decision regret score. Similar to the regret score, respondents were asked to what degree (on a 5‐point Likert scale) they agreed or disagreed with selected items taken from a decision‐making style instrument developed by Scott and Bruce^25^ (see Cerully et al.^22^ for details of the scale items selected). These responses are aggregated to create scores for each of four decision‐making styles: avoidant, dependent, intuitive and rational. Decision‐making skill was assessed using four decision tasks adapted from Bruine de Bruin et al.,^26^ presenting respondents with a hypothetical scenario of advising a friend on the purchase of a DVD player based on a quantitative symbolic representation of feature ratings. Each item tested skill in one of four decision rules (lexicographic, elimination by aspects, equal weights and satisficing), and responses were aggregated to create a decision‐making skill score.

The independent variables are presented in Table 2. They are divided into two categories: the covariates of interest and control variables.

**Table 2:** Independent variables classified as covariates of interest and controls

Variable	Description
*Covariates of interest*:	
Regret decisions	Tendency to experience doubt over decisions made (sum of five scale items, standardized)
Avoidant decision style	Evades decision‐making (sum of two decision‐style scale items, standardized)
Dependent decision style	Relies on guidance from others when making decisions (sum of three decision‐style scale items, standardized)
Intuitive decision style	Trusts gut when deciding (sum of two decision‐style scale items, standardized)
Rational decision style	Thoroughly searches for and logically evaluates alternatives (sum of two decision‐style scale items, standardized)
Decision‐making skill	Ability to apply specific decision rules (sum score from four choice tasks, standardized)
*Control variables*:	
Experimental arm	Seven randomly assigned arms varying on the type of data displayed (quantitative measures only, or also qualitative reviews); level of data (roll‐up scores only or drill‐down data); and formatting of data display. Arm 7 provided participants with a live ‘navigator’ via telephone to assist the user in navigating the website
Age	Respondent age
Education	Median year from each of 14 education categories
Gender	Female vs. male
Race	Black non‐Hispanic; Hispanic; Other non‐Hispanic; and 2+ races non‐Hispanic vs. White non‐Hispanic
Partnered	Married/living with partner vs. not (including divorced, never married, separated or widowed)
Income	Median value from each of 19 income categories, converted into log scale
Working	Working (including as a paid employee or self‐employed) vs. not working (including disabled, looking for work, on temporary layoff, retired, other)
Disabled	Disabled vs. not
Health	Excellent; very good; good; fair; poor
Activation	A cumulative score of six health consumer activation scale items, standardized
Prior exposure	Yes vs. no/not sure response to: In the past 12 months, do you remember seeing any information comparing different doctors, hospitals or health plans?

### 2.2 Analytic approach

For each of the 1247 consumer respondents, a rating for 10 doctor attributes is assigned once before exposure to doctor quality information and again after exposure. Ratings take the value of 1, 2 or 3. Since attributes are measured before and after exposure, we can define outcome variables as the differences calculated by subtracting the before rating from the after rating for each, which yields possible values of −2, −1, 0, 1 or 2. There are 10 outcome variables in total, each corresponding to a difference for a certain attribute. Changes in attribute valuations following exposure to doctor quality information are initially tested using standard exploratory techniques applied to the differences, including the Wilcoxon signed rank test.

Preliminary univariate probit regression models demonstrate that regression residuals from the individual probits are strongly correlated. Reliance on univariate models is not adequate in this case as they do not take into account this correlation between the outcomes and standard errors are unrealistically small. Therefore, to improve the reliability of the inferences and avoid misinterpretation, a multivariate approach is applied. Table A1 of the appendix demonstrates the correlation between the latent errors of the 10 attributes generated from the multivariate model. All the correlations are significantly different from zero, indicating that the attribute outcomes share common information and that a multivariate model is most appropriate.

To assess the impact of the covariates of interest on the categorical outcomes, a multivariate ordered probit regression model is considered. For example, see Varin and Czado^27^ and Hirk et al.^28^ for details on this model and inference methods. In the multivariate ordered probit model, the response differences are further compressed to take values of −1, 0 or 1 to obtain a parsimonious model in terms of the number of parameters and keep the computational burden at a manageable level. The attribute differences are regressed on the covariates of interest, which are measures of decision regret, decision‐making style and skill as well as control variables including respondent characteristics (age, education, gender, race/ethnicity, partner status, household income, employment, disability status and health status), the treatment arm assigned, level of activation and whether the respondent had past exposure to quality information. The individual characteristics included as controls are known to be related to decision‐making. Treatment arm is an important control as respondents were shown different displays of the quality measures and this could affect shifting valuations. Level of activation is another psychological construct thought to affect patient preferences, so is also included as a control to isolate the effects of the covariates of interest.^23^ Also, whether respondents had previously been exposed to quality information is included to control for differences that may result from different levels of experience with quality information.

The main focus of inference for this model is on the regression coefficients corresponding to each covariate. Similar to the standard probit regression model, such coefficients represent the relationship between covariates and the categorical responses. In the present model formulation, each attribute difference response corresponds to a set of specific regression parameters. Each response variable has its own set of regression parameters as shown in the columns reported in Tables B1 and C1 of the appendix. While the measurements on the 1247 respondents are assumed to be independent, in the multivariate probit regression model, dependence between attributes is modelled through the dependent regression errors, which are assumed to follow a multivariate normal distribution with zero mean and a 10×10 correlation matrix to be estimated. For this kind of model, estimation via maximum likelihood is not trivial for the case where there are 10 response variables. As an alternative, the composite likelihood estimation approach of Hirk et al.^28^ recently implemented in the R package mvord is applied to obtain the estimates reported in Tables B1 and C1 of the appendix.

## 3 RESULTS

**Figure 1:**
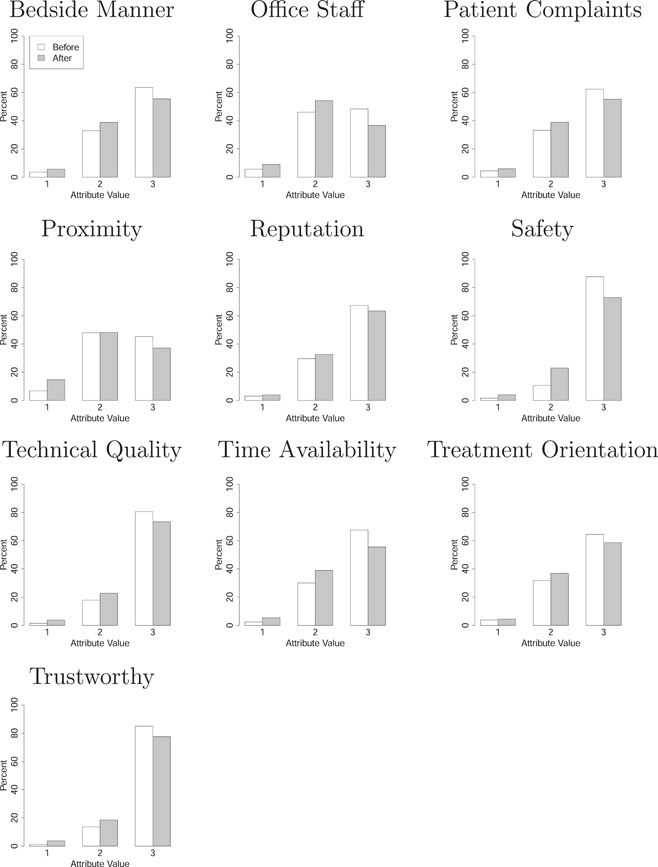
Distribution of the attribute valuations for selecting primary care doctors before and after exposure. Response options were as follows: 1 = not matter much; 2 = matter some; and 3 = matter a lot. The Wilcoxon signed rank test was used to determine the differences between before and after ratings. Tests for all 10 attributes were statistically significant, with *p*‐values <.01

Initial exploratory analyses indicate that respondents do in fact shift their valuation of doctor attributes following exposure to performance information. Figure 1 shows respondent valuation of the 10 attributes before and after exposure. The *x*‐axis shows the 1, 2 or 3 rating assigned to the attribute, with the white bar showing the before rating and the grey bar showing the after rating. The *y*‐axis shows the percentage of respondents assigning each rating. The results of the Wilcoxon signed rank test show that there is a statistically significant change in the distribution between before and after ratings for each of the 10 attributes, with *p*‐values smaller than .01 for all tests.

**Figure 2:**
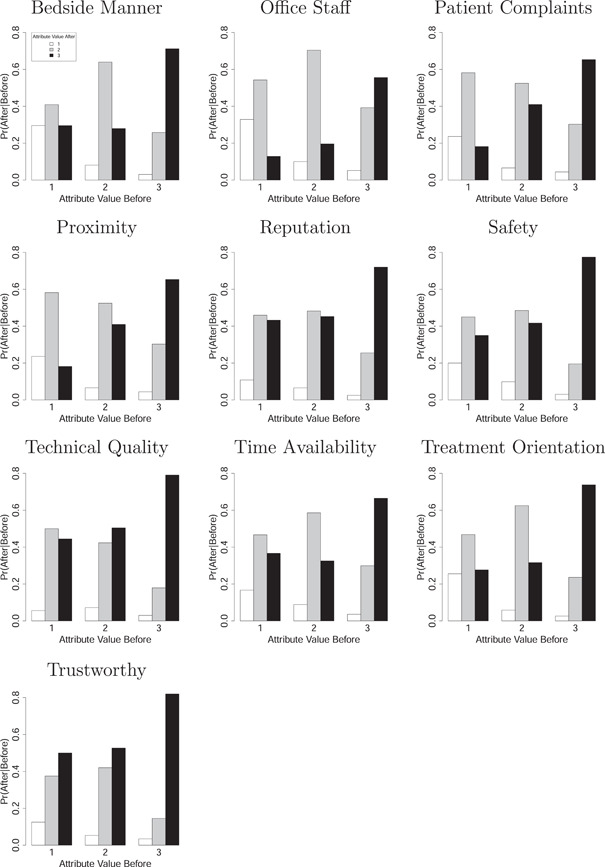
Estimated conditional probability of after valuations of attributes given before valuations

The pattern of these shifts differs depending on how respondents first rated the attribute. Figure 2 shows the estimated conditional probability of after valuations conditioned on before valuations. The *x*‐axis is the 1, 2 or 3 rating assigned to the attribute before exposure to performance information. The *y*‐axis shows the estimated probability of a post‐exposure rating given the pre‐exposure rating, with the white bar representing a rating of 1, the grey bar representing a rating of 2 and the black bar representing a rating of 3. The results show that for all attributes when valued first at a 3, or mattering a lot, the probability of maintaining a rating of 3 after exposure to performance information is markedly high. However, for those initially rating an attribute as a 1, or does not matter much, different patterns start to emerge. As a pointed example, technical quality shows the greatest abandonment of the lowest rating postexposure, with only a probability of .05 of remaining with a 1 rating and .50 and .44 probabilities of increasing to a 2 or 3 rating, respectively.

Table 3 shows the results from the multivariate ordered probit regression model for the covariates of interest. Full regression results are shown in Tables B1 and C1 of the appendix. There is a statistically significant association between being more likely to regret decisions and increasing one's valuation of both the safety (*p*‐value <.01) and technical quality (*p*‐value <.05) attributes following exposure to the quality information.

**Table 3:** Estimated regression coefficients (with standard errors in parentheses) for the decision‐making regret, style and skill covariates of interest from the multivariate ordered probit model of doctor attribution valuation changes

	Bedside manner	Office staff	Patient complaints	Proximity	Reputation	Safety	Technical quality	Time availability	Treatment orientation	Trustworthy
Regret decisions	0.024	−0.017	0.039	−0.016	−0.017	0.162	0.110	−0.024	−0.010	0.068
	(0.055)	(0.050)	(0.049)	(0.048)	(0.050)	(0.057)**	(0.053)*	(0.053)	(0.051)	(0.057)
Avoidant decision style	0.032	−0.012	−0.040	0.025	0.010	−0.087	−0.065	−0.073	−0.002	−0.038
	(0.052)	(0.051)	(0.049)	(0.049)	(0.050)	(0.055)	(0.056)	(0.051)	(0.052)	(0.058)
Dependent decision style	−0.070	−0.037	0.004	0.021	−0.126	0.018	−0.041	−0.041	0.021	−0.030
	(0.050)	(0.046)	(0.046)	(0.043)	(0.047)**	(0.053)	(0.050)	(0.047)	(0.051)	(0.053)
Intuitive decision style	−0.019	−0.049	0.008	−0.003	−0.022	0.004	−0.002	−0.015	−0.019	−0.032
	(0.045)	(0.043)	(0.044)	(0.041)	(0.044)	(0.047)	(0.044)	(0.045)	(0.044)	(0.046)
Rational decision style	0.047	−0.064	0.001	−0.066	0.018	0.001	−0.003	−0.029	0.000	0.052
	(0.049)	(0.044)	(0.046)	(0.044)	(0.046)	(0.051)	(0.045)	(0.046)	(0.048)	(0.048)
Decision‐making skill	−0.119	−0.013	0.064	−0.038	0.053	−0.094	−0.051	−0.071	−0.040	−0.083
	(0.043)**	(0.041)	(0.043)	(0.040)	(0.043)	(0.046)*	(0.047)	(0.043)	(0.043)	(0.046)

** *p*‐value <.01

* *p*‐value <.05.

Of the four decision‐making styles elicited in the postexperiment survey, the dependent decision‐making style is the only one that showed a statistically significant association with one of the outcome variables. There is an association between being a more dependent‐type decision‐maker and decreasing one's valuation of the reputation attribute (*p*‐value <.01) following exposure. Having a higher level of decision‐making skill is associated with decreasing one's valuation of both the bedside manner (P *p*‐value <.01) and safety (*p*‐value <.05) attributes.

## 4 DISCUSSION

Consumerism in health care is a growing phenomenon that has not yet reached its optimal state. In the broadest sense, this means that until we fully realize ‘people proactively using trustworthy, relevant information and appropriate technology to make better‐informed decisions about their health care options’,^29^ more work is left to be done. Large‐scale investments continue to be made into the development of public reports on health care provider performance in several countries. Also, policy makers in many more countries are also interested in fostering consumer choice among health care providers. Yet, much still remains unknown about consumer behaviour when it comes to making health care choices, in particular, the decision‐making process when selecting one's primary care doctor; this has been noted as an important ‘black box’ requiring more research to illuminate.^19^

The findings of the present study suggest that consumers may value doctor attributes differently after being exposed to doctor‐level performance measures. In general, this finding is in line with the literature, most often in the areas of health plan and hospital choice, which shows that consumers' perspectives can be influenced by performance reports.^30^, ^31^, ^32^ For example, Emmert and Schlesinger^31^ found that 80% of users reported being influenced by publicly reported hospital report cards. Hibbard et al.^32^ found that those who recalled seeing a comparative health plan report perceived the reported items to be more important in health plan selection compared to those who had not seen the report.

More specifically, the results of this study indicate that while people have the potential to learn from novel performance information, this appears to be associated, to some extent, with decision‐making regret, styles and skill. Schlesinger et al.^19^ extensively explored the ways in which these decision‐making characteristics affect processing of quality information, especially as complexity increases, based on data collected in the SelectMD 1.0 project. In terms of specific decision‐making styles, respondents were affected differently by more complex choice sets. For instance, respondents identified as maximizers, who made good‐quality choices within simpler choice sets, became much more confounded compared to other types of decision‐makers within more complex choice sets where patient comments were introduced, although not when this more complex choice set then doubled the number of physicians from 12 to 24.^19^ Instead, in the most complex choice set, it was choice avoiders who made the poorest‐quality decisions.^19^ Regarding decision‐making skill, Martino et al.^18^ found that consumers with better decision‐making skill were more likely to consider the full breadth of performance measures available compared to low‐skilled decision‐makers.

The findings of the current study help to build on these previous findings to show that audience decision‐making characteristics can have a critical impact on the level to which consumers absorb, process and ultimately utilize performance data. As hypothesized, the safety and technical quality attributes do appear to become more salient following exposure to the quality‐reporting website (Hypothesis 1); however, this phenomenon is prevalent for those who score higher on the regret scale, as it is these respondents who are more likely to upgrade their rating of these two attributes post‐exposure (Hypothesis 2). It seems that ‘regretters’ are particularly sensitive to data showing variance in doctors' performance in these areas. This finding highlights an important distinction from the extant literature, which has focused more on how the tendency to regret leads to delays or avoidance of decision‐making.^19^, ^33^ The current findings suggest that it may also motivate learning as another way to avoid regret is to become better informed.^34^ Because the experiment essentially forced respondents into making a choice, avoidance was less possible. This is not unlike many health care situations where people must make a timely decision about a treatment option. Therefore, when avoidance is more difficult, for those leaning towards decision regret, it may be that greater attention to new information is the consequence.

The results demonstrate that the level of avoidant or intuitive decision‐making style is unrelated to change in valuation of the doctor attributes. These findings are therefore consistent with the original hypothesis of limited change associated with these two decision‐making styles (Hypothesis 3). Instead, the dependent decision‐making style is associated with a change in attribute rating. Those who score higher on the dependent decision‐making scale are more likely to downgrade their rating of the reputation attribute following exposure to quality information (Hypothesis 4). This suggests that those who may typically be more reliant on word‐of‐mouth and recommendations from others when making a doctor choice may tend to recalibrate the importance they placed on reputation when presented with other information on which to base their decision.

Similar to the avoidant and intuitive decision‐making styles, the results demonstrate that the level of rational decision‐making style is unrelated to change in valuation of the doctor attributes. This is contrary to what was expected (Hypothesis 5) and it may be that rational decision‐makers had already carefully weighed the importance of the safety and technical quality attributes even before exposure to the novel quality data.

Those who score higher on decision‐making skill were more likely to downgrade their rating of the bedside manner and safety attribute following exposure to quality information. The downgrading of the safety attribute is contrary to what was hypothesized (Hypothesis 6), and the downgrading of the bedside manner attribute was not anticipated. It seems that higher‐skilled decision‐makers were more likely to place high valuation on the bedside manner and safety attributes before exposure to the quality information and sought to adjust these ratings downward following exposure to other factors.

One limitation of this study is that data are used secondarily from an experimental study designed for a different research objective. Had the study been designed with the present research question in mind, survey questions may have been worded differently or even structured in another way. For instance, it may have been more instructive to ask respondents to rank order the ten attributes in terms of importance rather than rating each one on a Likert scale. This would have avoided the event where respondents assign all elements equal rating. The study design did make some allowance for this by asking any respondent who assigned the top rating to more than three attributes to select the three attributes that mattered most. However, these were not given in a rank‐order fashion. An analysis utilizing this top three question was tested, but it did not add any notable new information. Nevertheless, the study design as it stood allowed for a clear picture of shifting valuation of attributes following exposure to quality information, given the inclusion of the query in both the pre‐ and post‐survey.

Another limitation of the study is that the linkages between the attributes valuated by the respondents in the pre‐ and post‐surveys and the performance measures actually presented on the quality‐reporting website are not identical. While the performance measures are suggestive of the majority of attributes that were rated, they cannot be precisely matched one to one, and some attributes had no corresponding measure available (i.e., patient complaints and treatment orientation). The performance measures reported on the website were grouped into three categories: Use of Effective Treatments (which can be broadly mapped to the technical quality attribute), Methods to Reduce Medical Errors (which can be broadly mapped to the safety attribute) and Patient Survey Results (broadly mapping to bedside manner, office staff, reputation, time availability and trustworthy). The proximity attribute is an outlier in the sense that the website was designed so that all doctors in the choice set were displayed as meeting the respondent's distance requirements, thus making this factor moot in the doctor choice task. Despite this limitation, the study design still allowed for new insight into how people shift their perspective on what factors are important in choosing a doctor after being presented with a website reporting doctor‐level performance information.

The findings of this study offer a greater understanding of how exposure to performance reports potentially informs and influences consumer valuation of doctor attributes. To the author's knowledge, this is the first study to demonstrate that presenting consumers with doctor‐level quality information can impact consumers' expressed preferences concerning doctor attributes. This study also demonstrates that consumers are not all alike and that the audiences for performance‐reporting websites may have different needs and preferences when consuming this information based on cognitive characteristics, such as those concerning decision‐making. These findings contribute to a growing evidence base to guide policy makers in the development of more relevant performance data for consumers faced with the decision of choosing a primary care doctor. Further research is needed to understand the connection between the preferences that consumers have for certain doctor attributes, their individual decision‐making tendencies and how doctor‐level performance information on these attributes can help consumers find the doctor who best meets their wants and needs.

## 6 FUNDING INFORMATION

This research study received no specific grant from any funding agency. The author is supported by a doctoral student scholarship provided by the Free University of Bozen‐Bolzano.

## 7 CONFLICT OF INTEREST

The author declares no conflict of interest associated with this research study.

## Data Availability

Research data are not shared.

## APPENDIX A

**Table A1:** Estimated latent error correlation matrix

	Bedside manner	Office staff	Patient complaints	Proximity	Reputation	Safety	Technical quality	Time Availability	Treatment Orientation	Trustworthy
Bedside manner	1.00	–	–	–	–	–	–	–	–	–
Office staff	0.39***	1.00	–	–	–	–	–	–	–	–
Patient complaints	0.17***	0.19***	1.00	–	–	–	–	–	–	–
Proximity	0.15***	0.21***	0.11*	1.00	–	–	–	–	–	–
Reputation	0.26***	0.21***	0.26***	0.14**	1.00	–	–	–	–	–
Safety	0.28***	0.18***	0.23***	0.20***	0.13**	1.00	–	–	–	–
Technical quality	0.20***	0.19***	0.19***	0.17***	0.21***	0.47***	1.00	–	–	–
Time availability	0.34***	0.31***	0.21***	0.17***	0.15***	0.29***	0.32***	1.00	–	–
Treatment orientation	0.23***	0.12**	0.15***	0.09*	0.16***	0.37***	0.40***	0.23***	1.00	–
Trustworthy	0.36***	0.29***	0.24***	0.12*	0.32***	0.45***	0.44***	0.31***	0.42***	1.00

*** *p*‐value <.001

** *p*‐value <.01

* *p*‐value <.05.

## APPENDIX B

**Table B1:** Estimated regression coefficients (with standard errors in parentheses) and thresholds from the multivariate ordered probit model of doctor attribution valuation changes, first five attributes

	Bedside manner	Office staff	Patient complaints	Proximity	Reputation
*Covariates of interest*:					
Regret decisions	0.024 (0.055)	−0.017 (0.050)	0.039 (0.049)	−0.016 (0.048)	−0.017 (0.050)
Avoidant decision style	0.032 (0.052)	−0.012 (0.051)	−0.040 (0.049)	0.025 (0.049)	0.010 (0.050)
Dependent decision style	−0.070 (0.050)	−0.037 (0.046)	0.004 (0.046)	0.021 (0.043)	−0.126 (0.047)**
Intuitive decision style	−0.019 (0.045)	−0.049 (0.043)	0.008 (0.044)	−0.003 (0.041)	−0.022 (0.044)
Rational decision style	0.047 (0.049)	−0.064 (0.044)	0.001 (0.046)	−0.066 (0.044)	0.018 (0.046)
Decision‐making skill	−0.119 (0.043)**	−0.013 (0.041)	0.064 (0.043)	−0.038 (0.040)	0.053 (0.043)
*Control variables*:					
Arm (vs. 1)					
2	−0.201 (0.163)	−0.149 (0.167)	0.042 (0.152)	0.004 (0.149)	−0.040 (0.153)
3	−0.010 (0.164)	−0.217 (0.160)	−0.099 (0.153)	−0.038 (0.143)	−0.291 (0.147)*
4	−0.009 (0.156)	−0.216 (0.155)	0.109 (0.150)	0.032 (0.149)	0.082 (0.147)
5	−0.125 (0.165)	−0.287 (0.163)	0.039 (0.157)	−0.089 (0.153)	0.020 (0.153)
6	−0.053 (0.156)	−0.121 (0.159)	−0.036 (0.155)	−0.063 (0.146)	−0.139 (0.157)
7	−0.053 (0.159)	−0.250 (0.155)	−0.125 (0.163)	−0.122 (0.153)	−0.097 (0.156)
Age	0.001 (0.003)	0.003 (0.003)	−0.002 (0.003)	−0.002 (0.003)	−0.002 (0.003)
Education	0.014 (0.020)	0.017 (0.020)	−0.005 (0.020)	−0.002 (0.020)	0.004 (0.019)
Gender (vs. male) female	−0.100 (0.091)	−0.033 (0.088)	−0.097 (0.087)	0.039 (0.087)	−0.070 (0.086)
Race (vs. white non‐HS)					
Black, non‐HS	−0.008 (0.179)	−0.014 (0.177)	−0.061 (0.170)	−0.116 (0.171)	−0.047 (0.163)
Hispanic	−0.073 (0.144)	−0.134 (0.159)	−0.043 (0.142)	0.081 (0.163)	0.107 (0.151)
Other, non‐HS	0.083 (0.249)	0.133 (0.263)	0.377 (0.269)	−0.106 (0.240)	0.127 (0.334)
2+ races, non‐HS	−0.301 (0.244)	−0.302 (0.234)	0.190 (0.245)	−0.074 (0.290)	−0.143 (0.230)
Partnered (vs. not) married/living with	−0.179 (0.102)	−0.210 (0.097)*	−0.104 (0.098)	−0.092 (0.096)	−0.049 (0.095)
Log. income	−0.038 (0.063)	0.019 (0.063)	0.090 (0.060)	0.068 (0.063)	−0.009 (0.062)
Working (vs. not) working	0.148 (0.101)	0.046 (0.097)	−0.073 (0.092)	0.023 (0.092)	0.066 (0.096)
Disabled (vs. not) disabled	−0.148 (0.236)	−0.130 (0.244)	−0.177 (0.202)	0.139 (0.202)	−0.257 (0.227)
Health (vs. excellent)					
Very good	0.061 (0.146)	−0.086 (0.141)	−0.075 (0.141)	0.237 (0.135)	0.049 (0.145)
Good	0.065 (0.153)	−0.056 (0.147)	−0.064 (0.144)	0.168 (0.142)	0.128 (0.152)
Fair	0.216 (0.209)	−0.074 (0.206)	−0.208 (0.191)	0.327 (0.204)	0.250 (0.198)
Poor	0.527 (0.353)	0.505 (0.411)	−0.189 (0.321)	0.493 (0.301)	0.266 (0.424)
Activation	0.001 (0.047)	0.005 (0.045)	−0.009 (0.043)	−0.020 (0.047)	0.011 (0.047)
Prior exposure (vs. yes)					
No	−0.074 (0.095)	0.030 (0.090)	−0.054 (0.087)	0.029 (0.089)	−0.025 (0.092)
Not sure	−0.010 (0.135)	0.041 (0.135)	0.050 (0.130)	−0.017 (0.142)	−0.027 (0.134)
${\hat{\zeta }}_{k1}$	−1.133 (0.747)	−0.420 (0.721)	−0.137 (0.697)	0.179 (0.697)	−1.00 (0.695)
${\hat{\zeta }}_{k2}$	0.925 (0.747)	1.412 (0.723)	1.543 (0.699)*	1.817 (0.698)**	0.853 (0.695)

** *p*‐value <.01

* *p*‐value <.05.

## APPENDIX C

**Table C1:** Estimated regression coefficients (with standard errors in parentheses) and thresholds from the multivariate ordered probit model of doctor attribution valuation changes, remaining five attributes

	Safety	Technical quality	Time availability	Treatment orientation	Trustworthy
*Covariates of interest*:					
Regret decisions	0.162 (0.057)**	0.110 (0.053)*	−0.024 (0.053)	−0.010 (0.051)	0.068 (0.057)
Avoidant decision style	−0.087 (0.055)	−0.065 (0.056)	−0.073 (0.051)	−0.002 (0.052)	−0.038 (0.058)
Dependent decision style	0.018 (0.053)	−0.041 (0.050)	−0.041 (0.047)	0.021 (0.051)	−0.030 (0.053)
Intuitive decision style	0.004 (0.047)	−0.002 (0.044)	−0.015 (0.045)	−0.019 (0.044)	−0.032 (0.046)
Rational decision style	0.001 (0.051)	−0.003 (0.045)	−0.029 (0.046)	0.000 (0.048)	0.052 (0.048)
Decision‐making skill	−0.094 (0.046)*	−0.051 (0.047)	−0.071 (0.043)	−0.040 (0.043)	−0.083 (0.046)
*Control variables*:					
Arm (vs. 1)					
2	−0.062 (0.178)	−0.041 (0.170)	−0.212 (0.164)	−0.085 (0.166)	0.036 (0.168)
3	−0.183 (0.161)	−0.117 (0.164)	−0.247 (0.154)	−0.221 (0.156)	−0.162 (0.169)
4	0.077 (0.175)	0.058 (0.169)	−0.158 (0.154)	−0.093 (0.159)	0.234 (0.166)
5	−0.042 (0.162)	0.076 (0.162)	−0.098 (0.157)	0.004 (0.167)	0.123 (0.164)
6	−0.068 (0.160)	−0.006 (0.159)	−0.285 (0.153)	−0.334 (0.157)*	−0.064 (0.159)
7	−0.031 (0.176)	0.321 (0.168)	−0.118 (0.157)	−0.036 (0.161)	0.065 (0.176)
Age	0.006 (0.003)	0.007 (0.003)*	0.001 (0.003)	0.003 (0.003)	0.006 (0.003)
Education	−0.067 (0.023)**	−0.014 (0.021)	−0.019 (0.020)	−0.012 (0.019)	−0.018 (0.022)
Gender (vs. male) female	0.040 (0.098)	−0.106 (0.093)	−0.047 (0.088)	−0.037 (0.088)	0.050 (0.096)
Race (vs. white non‐HS)					
Black, non‐HS	0.256 (0.225)	−0.142 (0.209)	−0.039 (0.176)	0.177 (0.180)	−0.013 (0.200)
Hispanic	0.052 (0.178)	−0.128 (0.166)	−0.086 (0.158)	−0.137 (0.179)	−0.173 (0.184)
Other, non‐HS	0.595 (0.289)*	0.217 (0.283)	0.358 (0.238)	−0.155 (0.234)	0.403 (0.276)
2+ races, non‐HS	0.207 (0.259)	−0.496 (0.374)	−0.173 (0.220)	−0.391 (0.294)	0.091 (0.276)
Partnered (vs. not) married/living with	−0.134 (0.108)	−0.160 (0.104)	0.006 (0.098)	0.051 (0.101)	−0.139 (0.110)
Log. income	0.066 (0.071)	0.067 (0.069)	0.070 (0.064)	0.056 (0.066)	−0.071 (0.070)
Working (vs. not) working	−0.137 (0.111)	0.098 (0.103)	0.006 (0.097)	−0.126 (0.101)	0.101 (0.109)
Disabled (vs. not) disabled	−0.258 (0.237)	−0.139 (0.234)	0.046 (0.230)	−0.038 (0.246)	−0.240 (0.250)
Health (vs. excellent)					
Very good	−0.017 (0.158)	−0.086 (0.142)	−0.018 (0.136)	0.105 (0.145)	−0.051 (0.159)
Good	−0.075 (0.166)	−0.077 (0.150)	0.024 (0.143)	−0.070 (0.153)	−0.054 (0.164)
Fair	−0.234 (0.233)	0.035 (0.214)	0.072 (0.203)	0.012 (0.196)	−0.133 (0.249)
Poor	0.077 (0.367)	−0.076 (0.322)	0.308 (0.371)	0.278 (0.420)	0.134 (0.359)
Activation	0.066 (0.046)	−0.033 (0.046)	0.015 (0.046)	0.008 (0.046)	−0.028 (0.048)
Prior exposure (vs. yes)					
No	0.020 (0.097)	−0.150 (0.095)	0.001 (0.090)	0.065 (0.092)	0.060 (0.098)
Not sure	0.119 (0.151)	0.002 (0.148)	0.047 (0.134)	0.090 (0.139)	0.096 (0.149)
${\hat{\zeta }}_{k1}$	−0.969 (0.843)	−0.297 (0.808)	−0.259 (0.733)	−0.423 (0.768)	−1.762 (0.802)*
${\hat{\zeta }}_{k2}$	1.512 (0.852)	1.940 (0.816)*	1.611 (0.733)*	1.622 (0.770)*	0.693 (0.801)

** *p*‐value <.01

* P *p*‐value <.05.
